# Assessment of morbidity in gynaecologic oncology laparoscopy and identification of possible risk factors

**DOI:** 10.3332/ecancer.2015.606

**Published:** 2015-12-14

**Authors:** Maite Peña-Fernández, Inés Solar-Vilariño, María Xosé Rodríguez-Álvarez, Ignacio Zapardiel, Francisco Estévez, Pilar Gayoso-Diz

**Affiliations:** 1 Department of Gynecology and Obstetrics, Santiago de Compostela University Clinical Teaching Hospital, Spain; 2 Clinical Epidemiology and Biostatistics Unit, Santiago de Compostela University Clinical Teaching Hospital and Santiago Health Research Institute, Spain; 3 Gynecologic Oncology Unit, La Paz University Hospital, Madrid, Spain; 4 Department of Gynecology and Obstetrics, POVISA Hospital, Vigo, Spain

**Keywords:** cervical cancer, endometrial cancer, complications, laparoscopy

## Abstract

**Background:**

The aim of this study was to ascertain the incidence of and the risk factors associated with morbidity in laparoscopy performed on patients with cervical cancer and endometrial cancer.

**Methods:**

This was an observational study of a cohort of 128 women, 89 with endometrial cancer and 39 with cervical cancer from January 2000 to December 2011. We used the Student’s t-test or the Mann-Whitney U test for continuous variables, and the Chi-square or Fisher’s exact test for categorical variables.

**Results:**

Complications were found in 44 patients (34.4%). After a multivariate analysis, among the risk factors associated with the presence of complications as the only type of surgery was found to be statistically significant (*p* = 0.043), more frequent in the most complex procedures such as Wertheim operation, trachelectomy, and para-aortic lymphadenectomy. Type of surgery (*p* = 0.003) and tumour type (*p* = 0.003) were risk factors associated with conversion to laparotomy. It was more frequent among the most complex procedures and cervical cancer cases. Regarding the need for transfusion, significant differences were observed in terms of surgery duration (*p* < 0.001), more frequent in longer surgery.

**Conclusion:**

Morbidity in laparoscopic surgical oncology is related to the surgery complexity, where the basal characteristics of the patient are not a factor of influence in the development of complications.

## Introduction

The most frequent gynaecologic tumour is endometrial cancer (EC) followed by cervical cancer (CC). Every year 198,000 new cases of EC [1] and 500,000 new cases of CC are diagnosed around the world, this being 83% in developed countries [2].

In many patients who suffer from these types of tumours, surgery forms part of their treatment. Because of their baseline disease, these cases involve special operative morbidity.

Indeed, it was precisely this goal of reducing operative morbidity and ensuring a better quality of life that led to the development of laparoscopic surgery for these tumours. A number of studies have demonstrated the safety and the effectiveness of this procedure as compared to conventional laparotomy, with evidence to show that laparoscopy results in a shorter hospital stay, lower morbidity, and the same survival rate [3–6]. In the case of EC, it should be noted that the Gynaecologic Oncology Group’s LAP2 study showed that at three years there were no differences in overall survival (OS) among patients who had undergone laparoscopic or laparotomic surgery (89.8% versus 89.9%) [7]; and in the case of CC, a follow-up study of laparoscopy patients reported 92.7% OS and 91.1% disease-free survival (DFS) at 7.2 years [8].

Our study aimed at ascertaining the complications inherent in this type of patients in view of the fact that, while laparoscopy is widely reputed to be a safe surgical technique with a very low percentage (0–10%) of complications [9–11], the relevant data have nevertheless been drawn from different series which have largely included benign surgical processes. We thus thought it would be of interest to ascertain the incidence of complications in laparoscopy performed in oncologic patients who present with greater morbidity owing to the surgical difficulty entailed and their baseline disease. Moreover, this subject has been addressed by few studies directly.

## Methods

We conducted an observational study on a cohort of EC and CC patients who underwent laparoscopy at the Gynaecology Department of the POVISA Hospital, (Vigo, Spain), from January 2000 until December 2011. This study was undertaken with the approval of the Galician Clinical Research Ethics Committee. Excluded from the study were: patients who did not undergo monitoring at the above health centre, defined as a three month follow-up period for study assessment purposes; and those whose clinical history did not allow collection of data on the study variables.

The variables were retrospectively collected by a review of clinical histories. We analysed patient characteristics, such as age, pre- and postoperative haemoglobin (Hb) in g/dL, body mass index (BMI; patient’s weight in kilograms divided by height in meters squared), previous abdominal surgery, and comorbidities (arterial hypertension, diabetes mellitus, thyroid disorders, chronic treatment with corticosteroids or immunomodulatory drugs). In the case of tumour characteristics, we recorded histologic type, grade, Federation of Obstetricians and Gynaecologists (FIGO) stage, and lymph node involvement. Types of surgery are stratified depending on the level of complexity: Level 1 (hysterectomy +/- double adnexectomy), level 2 (all surgery procedures with pelvic lymphadenectomy), and level 3 (Wertheim, trachelectomy and surgery procedures with para-aortic lymphadenectomy). Regarding the surgical technique used, the following variables were included: presence of adhesions, number of resected lymph nodes, and surgery duration (minutes). Surgeons’ experience was evaluated in years dating back from their first advanced laparoscopy. Complications were divided into intra- and postoperative (i.e. diagnosed in the first three months post-surgery), and included nerve, intestinal, urologic, and vascular lesions, ileum, lymphocysts, fistulae, bladder dysfunction, infection, haemoperitoneum, thromboembolic events, and trocar site hernia. Other data collected included the need for and cause of conversion to laparotomy, and the use of blood transfusions.

For the analysis of risk factors according to BMI, patients were divided into the following two groups: those with a BMI of less than 30; and those that presented with overweight and obesity, namely a BMI of 30 or over. We identified patients with postoperative anaemia, defined as any person who had a lower postoperative Hb of 11.5 g/dL, or who on comparing pre- and postoperative Hb levels registered a decline of 3 g/dL or more. Similarly, tumours were categorised as advanced or early FIGO stage, with early stage being defined as I–II in EC and as I–IIa in CC, and advanced stage being defined as III–IV in EC and as IIb–IV in CC.

### Statistical analysis

Continuous variables were expressed as the median (interquartile range) and categorical covariates as percentages. The association between each of the study variables with (a) type of cancer, (b) complications, (c) conversion to laparotomy, and (d) transfusion was statistically analysed using the Student’s t-test or the Mann-Whitney U test for continuous variables, and the Chi-square or Fisher’s exact test for categorical variables. Variables having a *p*-value of less than 0.2 in the univariate analysis were incorporated into a multivariate logistic regression model that also included age, regardless of its statistical significance in the univariate analysis. A stepwise procedure based on the Wald test was then used to select the best subset of explanatory risk factors, while maintaining type of cancer and age as possible confounding variables in the regression model. Results were expressed as Odds Ratios (ORs) along with their 95% confidence intervals (CI). All tests were two-sided, with *P* < 0.05 regarded as statistically significant. We performed all the analysis using the R software programme, version 2.15.1 [12].

## Results

A total of 128 patients were analysed, 89 with EC and 39 with CC. The characteristics of both groups are described in Table 1, which shows that these two groups are differentiated in terms of patient’s baseline characteristics and surgery type, but no differences in terms of FIGO stage and existence of previous surgery.

EC group is formed in majority by endometrioid adenocarcinoma cases i.e. a total of 78. The rest being two cases of serous adenocarcinoma, carcinosarcoma and adenocarcinoma mixed carcinoma (endometrioid and clear cell) and one case of polypoid carcinosarcoma, endometrial leiomyosarcoma, clear cell adenocarcinoma, papillar adenocarcinoma, and squamous adenocarcinoma. The distribution by FIGO stage is shown in Table 1. The histological grade (G) distribution is conformed by 28 G1, 34 G2, and 25 G3, and for only two cases we did not find data in their clinical histories.

Histological distribution CC group is formed by squamous carcinoma 22 cases, adenocarcinoma 12 cases, adenosquamous 2 cases, 1 neuroendocrine tumour, and 1 granulosa cell tumour. Table 1 shows the number of cases by FIGO stage. By grade we found, 4 G1, 11 G2 and 10 G3, and there were 14 cases without data available about that.

The type of surgery performed and its distribution according to surgeons’ experience are shown in Table 2. The median hospital stay was seven days.

There were complications in a total of 44 patients, 22 cases in EC, and 22 cases in CC, an overall incidence of 34.4%. Despite this morbidity percentage, the mortality showed in the follow-up was 0%.

The different types of complications were: intraoperative complications, which include nerve lesion, vascular lesion and urologic lesion; and postoperative complications of: obstructive uropathy, ileum, lymphocysts, fistulae, bladder dysfunction, infection, haemoperitoneum, thromboembolic disease, trocar site hernia, and other complications. Intraoperative complications were less frequent (19 cases); they mostly took the form of vascular lesions (12 cases). Postoperative complications (54 cases) were more heterogeneously distributed, with trocar site hernia and thromboembolic events being the most frequent, in eight and seven cases respectively. Under the header of ‘other complications’ were included: abdominal wall haematoma; respiratory distress syndrome; paroxysmal atrial fibrillation; pulmonary oedema; drain entrapment; trocar site tumour recurrence; rectus sheath haematoma; seroma; and pleural effusion. If we use the Clavien-Dindo grading system to classify postoperative complications, the distribution is as seen in Table 3. We find that the most frequent are type III i.e. those which need surgery consisting of 22 cases, majority of them because of trocar site hernia which amount to a total of eight cases. The second in frequency with five cases is obstructive uropathy. On the other side type IV, complications that need intermediate or intensive care included only six cases, five with total pulmonary thromboembolism. Those with minor complications which have a pharmacological solution, i.e. type I and II conformed as the majority group with 26 cases. [13].

After fulfilling the univariant analysis, the risk factors statistically significant in relation to the development of complications are: type of cancer, age, surgical time, and type of surgery, as shown in Table 4. When applying the multivariant analysis the only risk factor is type of surgery, complications being more frequent in more complex surgeries, level 3 (*p*-value 0.043). The type of complications related to surgical processes level 3 are: vascular lesion, fistulas, bladder dysfunction, and infection, showing statistical significance in Table 5.

Even though surgeons’ experience is not presented as a risk factor, when breaking down the complications according to the surgeons’ experience, Table 6, less experienced surgeons present a higher risk of hernia through the trocar, one of the most frequent postsurgical complications.

Studying specific risk factors, the influence of the number of lymph nodes extracted on the development of lymphocysts did not prove to be statistically significant. Patients with lymphocysts presented with a median of 13 resected lymph nodes compared with a median of 12.5 in the group of patients without lymphocysts (*p*-value 0.76).

Furthermore, a study of the relationship between the development of thromboembolic events on the one hand and duration of surgery and BMI on the other, showed that surgery in patients with thrombosis had a median duration of 170 as opposed to 185 minutes in those without thrombotic involvement, with no significant differences in evidence (*p*-value 0.8). In the case of BMI, a comparison of patients who presented thrombosis showed no statistically significant difference between those with a BMI of under 30 and those with a BMI of 30 or over, with the respective figures being 57.1% versus 42.9% (*p*-value 1.00).

The relation of the BMI is analysed with the development of vascular and urologic lesions, being in both cases not relevant, with p- value of 0.7 and 0.1 respectively.

The factors associated with the need for transfusion or conversion to laparotomy are analysed in Table 7. Regarding the transfusions, significant differences were observed in terms of type of cancer, with transfusion proving more frequent in CC than in EC, 59.9% versus 26.4%, in longer surgical procedures (254 minutes versus 169 minutes), and in type of surgery, more frequent in level 3, i.e. 54.3%. After applying the multivariate model, the only statistically significant factor was surgery duration (*p*-value <0.001).

The statistically significant risk factors for conversion to laparotomy were type of cancer and type of surgery, with conversion being more frequent in CC than in EC, 14.4% versus 2.2%, and in patients with surgery level 3 this being 85.7%. The mentioned factors could not been confirmed applying a multivariate model, since there were no cases of conversion in surgery level 2.

## Discussion

In our series, the incidence of complications is 34.4%. This might be regarded as a high figure compared with a previous paper [10] that analysed complications in a Gynaecologic Oncology Service and reported a 9% complication rate in 1451 laparoscopies. However, this group covered all types of interventions, including diagnostic and second-look laparoscopies, salpingectomies, etc. Among these there were only 79 high-level procedures, of which two were radical hysterectomies. Closer to our percentage of complications are other studies which addressed laparoscopy in EC [3, 4, 5, 6, 14] and reported complication rates ranging from 5–33%. It should however, be stressed here that whereas many of these studies covered laparoscopies which were mainly performed on stage-I cases, our sample also included 15 cases in stages III–IV. In the case of CC, there are fewer studies. By extension in one study with a smaller volume of patients; [8] i.e. with 139 cases of CC, reported a complication rate of 21.58%.

The percentage of conversions to laparotomy in our sample was 5.47%, which comes within the range cited by some randomised studies, i.e., 2.4–25.8% [6], with the highest figure, 25.8%, being reported by the LAP2 study. The risk factors related to the conversion to laparotomy are the type of surgery and the type of tumour, being more frequent in more complex surgeries and CC. Both factors seem to be related, since in all CC more complex surgeries are conducted. It should be underlined that in our sample, six out of seven cases of conversion to laparotomy were vascular lesions that could not be controlled through laparoscopy.

On seeking to identify possible risk factors for development of complications, significant differences were only observed with respect to surgery type, more frequent in level 3. Analysis of the distribution of the different types of complications in our sample showed that there were significant differences in terms of development of fistulae, bladder dysfunction, infection, and vascular lesions, with all of these being more frequent among women undergoing surgery level 3. Other publications support that the difficulty of surgical procedures is a risk factor in the development of complications [9], especially the laparoscopic radical hysterectomy is associated with a significant increased risk of intraoperative urologic complications [15]. Although there is a clear risk of urinary lesions during radical hysterectomy, this is not increased by laparoscopy. Instead, the percentage remains the same as for laparotomy, as shown by one study [16] which reported an intraoperative urologic lesion rate of 8% for laparoscopy versus 10.4% for laparotomy, and a postoperative urologic lesion rate of 14% for laparoscopy versus 22.9% for laparotomy.

A recent study related obesity with a higher risk of urologic lesion [15], which lead us to analyse if the BMI influences the urologic lesions, but no statistical significance was found (*p*-value = 0.7). BMI did not affect the subsequent development of complications, conversion to laparotomy and need transfusion, a finding in line with other publications [1, 17].

While states of anaemia have been associated with the development of postoperative infections [18], no causal relationship was observed in our sample because there were only five cases of infection. Nevertheless, all of these cases of infection occurred in anaemic patients.

Prolonged surgery durations have been related with nerve lesions [6], some hypotheses found in the literature regarding this are the patient’s exposure to anesthesia and the persistence of positions that can cause maintained compression. In our study there was only one nerve lesion hence no analysis could be performed in this respect. It has to be said, however, that this intervention lasted 125 minutes, very much below the mean surgery time for the total sample, which was 205 minutes.

The percentage of adhesions developed after abdominal surgery was in the order of 21% [19]. The presence of adhesions can hinder an intervention and so raise the risk of complications. Nevertheless, as Table 3 shows, neither the existence of previous surgery nor the presence of adhesions increased the risk of subsequent complications.

It should be pointed out that, in our sample, operations were performed on four patients who had previously received radio- and/or chemotherapy treatment, something that could increase operative morbidity. Even so, there are too few cases for any conclusion to be drawn in this respect. Of these four patients, only one presented a complication, with a vascular lesion at the level of the inferior epigastric artery.

The incidence of port-site metastasis was very low and stood at around 0.41–0.97% [20, 21]. Its presence has been related with more advanced cases of cancer or patients with worse prognosis, positive lymph nodes, carcinomatosis, or metastatic disease spread to other parts of the body. In our study there was only one case, which amounted to an incidence of 0.77%, involving a patient with a FIGO stage IB-G II endometrioid adenocarcinoma with negative lymph nodes.

To unify the sample, univariate and multivariate analysis were conducted excluding the high rank tumours and doing different analysis for cervix and endometrial cancer. This does not add any new interesting data to the original sample of 128 cases, where the risk factor for the complications and conversion to laparotomy is type of surgery and the risk factor for need of transfusion is surgery time.

Among the limitations of our study is the fact that it was based on a small number of cases and that it was retrospective. Despite these limitations, the results show that the radical nature of the type of intervention performed constituted the principal risk factor for development of complications and conversion to laparotomy. The patient’s basal characteristics (obesity, previous surgeries, etc.) do not imply a higher surgical morbidity. From this it can be inferred that in the case of oncologic patients, laparoscopic surgery is safe but that the specific intervention performed will dictate the final outcome.

## Conclusion

Morbidity in laparoscopic surgical oncology is related to the surgery complexity, where the basal characteristics of the patient are not a factor of influence on the development of complications.

## Figures and Tables

**Table 1.** Patient characteristics by type of cancer.

Characteristics	Type of cancer		*p*-value
	Endometrial (*n* = 89)	Cervical (*n* = 39)	
**Age**	64.0 [56.0; 71.0]	49.0 [39.0; 56.0]	<0.001
**BMI** ≤30 >30	45 (52.9%) 40 (47.1%)	32 (84.2%) 6 (15.8%)	0.002
**Comorbidities** No Yes	37 (41.6%) 52 (58.4%)	29 (74.4%) 10 (25.6%)	0.001
**Arterial hypertension** No Yes	52 (58.4%) 37 (41.6%)	34 (87.2%) 5 (12.8%)	0.003
**Diabetes mellitus** No Yes	76 (85.4%) 13 (14.6%)	38 (97.4%) 1 (2.6%)	0.063
**Thyroid disease** No Yes	76 (85.4%) 13 (14.6%)	38 (897.4%) 1 (2.6%)	0.063
**Corticosteroids** No Yes	98 (100%) 0 (0%)	37 (94.9%) 2 (5.1%)	0.091
**Immunomodulatory treatments** No Yes	88 (98.9%) 1 (1.1%)	34 (87.2%) 5 (12.8%)	0.010
**Previous abdominal surgery** No Yes	54 (60.7%) 35 (39.3%)	28 (71.8%) 11 (28.2%)	0.314
**FIGO stage** Early Advanced	77 (86.5%) 12 (13.5%)	33 (91.7%) 3 (8.3%)	0.551
**Surgery** Level 1 Level 2 Level 3	23 (25.8%) 59 (66.3%) 7 (7.87%)	4 (10.3%) 1 (2.5%) 34 (87.2%)	<0.001

**Table 2.** Overall distribution of types of surgery, according to surgeons’ experience.

Type of surgery	Number of cases (*n* = 128)	Surgeons’ experience in years	
		≤5 (*n* = 44)	>5 (*n* = 84)
Level 1^*^	27 (21.1%)	11 (25.0%)	16 (19.0%)
Level 2^†^	60 (46.9%)	20 (45.5%)	40 (47.6%)
Level 3^‡^	41 (32.0%)	13 (29.5%)	28 (33.3%)

* Hysterectomy +/- bilateral adnexectomy.

† Surgery with pelvic lymphadenectomy.

‡ Wertheim, trachelectomy, surgery with para-aortic lymphadenectomy.

**Table 3.** Postoperative complications distribution according Clavien-Dindo grading system.

Grade	Number complications (%)
I	16 (29.6%)
II	10 (18.5%)
III	22 (40.7%)
IV	6 (11%)
V	0 (0%)

**Table 4.** Risk factors and presence of complications.

Variable	Complications		*p*-value
	No (*n* = 84)	Yes (*n* = 44)	
**Type of cancer** Endometrial Cervical	67 (79.8%) 17 (20.2%)	22 (50%) 22 (50%)	0.001
**Age**	62.5 [51.0; 69.2]	56.0 [48.2; 62.5]	0.032
**Surgery duration**	172 [129; 230]	244 [155; 310]	0.006
**BMI** ≤30 >30	49 (61.3%) 31 (38.8%)	28 (65.1%) 15 (34.9%)	0.820
**Previous abdominal surgery** No Yes	54 (64.3%) 30 (35.7%)	28 (65.1%) 16 (34.9%)	1.000
**Adhesions** No Yes	66 (78.6%) 18 (21.4%)	38 (86.4%) 6 (13.6%)	0.404
**Comorbidities** No Yes	40 (47.6%) 44 (52.4%)	26 (59.1) 18 (40.9%)	0.295
**Smoking** Non-smoker Smoker	71 (84.5%) 13 (15.5%)	34 (77.3%) 10 (22.7%)	0.440
**Alcohol** Yes No	61 (72.6%) 23 (27.4%)	28 (63.6%) 16 (36.4%)	0.397
**FIGO Stage** Early Advanced	69 (85.2%) 12 (14.8%)	41 (93.2%) 3 (6.8%)	0.305
**Radio + Chemo** No Yes	81 (96.4%) 3 (3.6%)	43 (97.7%) 1 (2.3%)	1.000
**Surgeons’ experience** ≤five years >five years	33 (39.3%) 51 (60.7%)	11 (25.0%) 33 (75.0%)	0.156
**Type of surgery** Level 1 Level 2 Level 3	19 (22.6%) 48 (57.1%) 17 (20.2%)	8 (18.2%) 12 (27.3%) 24 (54.5%)	<0.001

**Table 5.** Complications according to type of surgery.

Complications	Type of Surgery			*p*-value
	Level 1 (*n* = 27)	Level 2 (*n* = 60)	Level 3 (*n* = 41)	
**Intraoperative**				
Nerve lesion No Yes	27 (100%) 0 (0.0%)	59 (98.3%) 1 (1.7%)	41 (100%) 0 (0.0%)	1.000
Vascular lesion No Yes	25 (92.6%) 2 (7.4%)	58 (96.7%) 2 (3.3%)	33 (80.5%) 8 (19.5%)	0.023
Intestinal lesion No	27 (100%)	60 (100%)	41 (100%)	
Urologic lesion No Yes	26 (96.3%) 1 (3.7%)	59 (98.3%) 1 (1.7%)	37 (90.2%) 4 (9.8%)	0.155
**Postoperative**				
Obstructive uropathy No Yes	27 (100%) 0 (0.0%)	59 (98.3%) 1 (1.7%)	37 (90.2%) 4 (9.8%)	0.107
Ileum No Yes	25 (92.6%) 2 (7.4%)	60 (100%) 0 (0.0%)	40 (97.6%) 1 (2.4%)	0.082
Lymphocysts No Yes	27 (100%) 0 (0.0%)	58 (96.7%) 2 (3.3%)	37 (90.2%) 4 (9.8%)	0.219
Fistulae No Yes	27 (100%) 0 (0.0%)	60 (100%) 0 (0.0%)	38 (92.7%) 3 (7.3%)	0.040
Bladder dysfunction No Yes	27 (100%) 0 (0.0%)	60 (100%) 0 (0.0%)	35 (85.4%) 6 (14.6%)	0.001
Infection No Yes	26 (93.6%) 1 (3.7%)	60 (100%) 0 (0.0%)	37 (90.2%) 4 (9.8%)	0.029
Haemoperitoneum No Yes	27 (100%) 0 (0.0%)	59 (98.3%) 1 (1.7%)	40 (97.6%) 1 (2.4%)	1.000
Thromboembolic disease No Yes	25 (92.6%) 2 (7.4%)	58 (96.7%) 2 (3.3%)	38 (92.7%) 3 (7.3%)	0.603
Trocar site hernia No Yes	25 (92.6%) 2 (7.4%)	58 (96.7%) 2 (3.3%)	37 (90.2%) 4 (9.8%)	0.452
Other complications No Yes	26 (93.6%) 1 (3.7%)	55 (91.7%) 5 (8.3%)	37 (90.2%) 4 (9.8%)	0.766

**Table 6.** Complications according to the surgeon’s experience.

Complications	Surgeon’s experience		*p*-value
	≤five years (*n* = 44)	>five years (*n* = 84)	
**Intraoperative**			
Nerve lesion No Yes	44 (100%) 0 (0.0%)	83 (98.8%) 1 (1.2%)	1.000
Vascular lesion No Yes	43 (97.7%) 1 (2.3%)	73 (86.9%) 11 (13.1%)	0.057
Intestinal lesion No	44 (100%)	84 (100%)	–
Urologic lesion No Yes	42 (95.5%) 2 (4.5%)	80 (95.2%) 4 (4.8%)	1.000
**Postoperative**			
Obstructive uropathy No Yes	41 (93.2%) 3 (6.8%)	82 (97.6%) 2 (2.4%)	0.338
Ileum No Yes	42 (95.5%) 2 (4.5%)	83 (98.8%) 1 (1.2%)	0.272
Lymphocysts No Yes	43 (97.7%) 1 (2.3%)	79 (94.0%) 5 (6.0%)	0.663
Fistulae No Yes	44 (100%) 0 (0.0%)	81 (96.4%) 3 (3.6%)	0.551
Bladder dysfunction No Yes	43 (97.7%) 1 (2.3%)	79 (94.0%) 5 (6.0%)	0.663
Infection No Yes	42 (95.5%) 2 (4.5%)	81 (96.4%) 3 (3.6%)	1.000
Haemoperitoneum No Yes	44 (100%) 0 (0.0%)	82 (97.6%) 2 (2.4%)	0.545
Thromboembolic disease No Yes	43 (97.7%) 1 (2.3%)	78 (92.9%) 6 (7.1%)	0.421
Trocar site hernia No Yes	38 (86.4%) 6 (13.6%)	82 (97.6%) 2 (2.4%)	0.020
Other complications No Yes	43 (97.7%) 1 (2.3%)	75 (89.3%) 9 (10.7%)	0.163

**Table 7.** Risk factors in transfusion and conversion to laparotomy.

	Transfusion		*p*-value	Conversion		*p*-value
Variable	No (*n* = 82)	Yes (*n* = 46)		No (*n* = 121)	Yes (*n* = 7)	
**Type of cancer** Endometrial Cervical	66 (80.5%) 16 (19.5%)	23 (50%) 23 (50%)	0.001	88 (72.7%) 33 (27.3%)	1 (14.3%) 6 (85.7%)	0.003
**Age**	61 [53; 68]	56 [44.8; 66.8]	0.177	60 [50; 68]	50 [39.5; 56.5]	0.069
**Surgery duration**	167 [125; 218]	248 [174; 335]	<0.001	180 [135; 250]	270 [225; 310]	0.064
**BMI** ≤30 >30	44 (56.4%) 34 (43.6%)	33 (73.3%) 12 (26.7%)	0.094	71 (60.7%) 46 (39.3%)	6 (100%) 0 (0.0%)	0.083
**Previous abdominal surgery** No Yes	54 (65.9%) 28 (34.1%)	28 (60.9%) 18 (39.1%)	0.710	77 (63.6%) 44 (36.4%)	5 (71.4%) 2 (28.6%)	1.000
**Adhesions** No Yes	67 (81.7%) 15 (18.3%)	37 (80.4%) 9 (19.6%)	1.000	98 (81.0%) 23 (19.0%)	6 (85.7%) 1 (14.3%)	1.000
**Comorbidities** No Yes	39 (47.6%) 43 (52.4%)	27 (58.7%) 9 (41.3%)	0.305	61 (50.4%) 60 (49.6%)	5 (71.4%) 2 (28.6%)	0.442
**Smoking** Non-smoker Smoker	68 (82.9%) 14 (17.1%)	37 (80.4%) 9 (19.6%)	0.910	101 (83.5%) 20 (16.5%)	4 (57.1%) 3 (42.9%)	0.109
**Alcohol** No Yes	56 (68.3%) 26 (31.7%)	33 (71.7%) 13 (28.3%)	0.837	83 (68.6%) 38 (31.4%)	6 (85.7%) 1 (14.3%)	0.675
**FIGO Stage** Early Advanced	70 (87.5%) 10 (12.5%)	40 (88.9%) 5 (11.1%)	1.000	104 (88.1%) 14 (11.9%)	6 (85.7%) 1 (14.3%)	1.000
**Radio + Chemo** No Yes	80 (97.6%) 2 (2.4%)	44 (95.7%) 2 (4.3%)	0.618	117 (96.7%) 4 (3.3%)	7 (100%) 0 (0.0%)	1.000
**Surgeons’ experience** ≤five years >five years	27 (32.9%) 55 (67.1%)	17 (37.0%) 29 (63.0%)	0.790	43 (35.5%) 78 (64.5%)	1 (14.3%) 6 (85.7%)	0.421
**Type of Surgery** Level 1 Level 2 Level 3	21 (25.6%) 45 (54.9%) 16 (19.5%)	6 (13.0%) 15 (32.6%) 25 (54.3%)	<0.001	26 (21.5%) 20 (49.6%) 35 (28.9%)	1 (14.3%) 0 (0.0%) 6 (85.7%)	0.003
